# Efficient elimination of *Yam mosaic virus* (YMV) from white yam (*Dioscorea rotundata* Poir.) by cryotherapy of axillary buds

**DOI:** 10.1016/j.sajb.2019.12.022

**Published:** 2020-05

**Authors:** Effiom Eyo Ita, Edak Aniedi Uyoh, Ikuo Nakamura, Valentine Otang Ntui

**Affiliations:** a Department of Genetics & Biotechnology, University of Calabar, Calabar, Nigeria; b Laboratory of Plant Cell Technology, Graduate School of Horticulture, Chiba University, Japan; c International Institute of Tropical Agriculture, Nairobi, Kenya

**Keywords:** Axillary buds, Cryotherapy, *Dioscorea rotundata*, *Yam mosaic virus*

## Abstract

*Dioscorea rotundata* is an economically important food crop in many tropical countries as many people in this region depend on it for food and livelihood. Viral diseases, especially *Yam mosaic virus* (YMV), constitute a major constraint in the cultivation of this crop as they perpetuate through generations in the vegetatively propagated planting materials. Getting resistant or at least virus-free planting materials for farmers thus becomes crucial. This study was aimed at eliminating YMV in *Dioscorea rotundata* by cryotherapy of axillary buds. Enlarged axillary buds of YMV-infected TDr 2269 were frozen in liquid nitrogen for 1 h, re-warmed at 40 °C and cultured to regenerate plantlets. Approximately 76.33% plantlet regeneration and 100% YMV eradication were obtained for cryo-treated buds, against 95% and 0% obtained respectively for non-treated buds. RT-PCR and RT-qPCR analyses did not reveal detectable quantity of YMV in treated plants but did in control plants. Plants from cryo-treated buds showed no mosaic symptoms and produced slightly more tubers, and heavier mini-tubers (20.48±3.11 g) under greenhouse conditions contrary to non-treated plants that showed severe mosaic symptoms with significantly smaller tubers (1.91±0.39 g) (*P* < 0.05). This is the first report showing the elimination of YMV from infected white yam stock plant by cryotherapy and would be useful for producing clean planting materials.

## Highlights

• Cryo-treatment of YMV-infected *D. rotundata* effectively eliminates YMV.
• No YMV can be detected in cryo-treated *D. rotundata in vitro* and ex vivo.
• Cryotreated YMV-infected *D. rotundata* do not show mosaic symptoms in greenhouse.
• Cryotreated YMV-infected *D. rotundata* produce healthier minitubers.

## 1 Introduction

The world's human population currently estimated at 7.7 billion is expected to exceed 9 billion by 2050 (Braun, 2010; UN, 2017). This will be driven by India and African countries, and Nigeria is expected to be the third largest with 411 million human population after China (1.3 billion) and India (1.6 billion) according to forecast by the 2017 revision of world population reports (UN, 2017). Such a rapidly increasing population will pose a great challenge for global food security. A drastic increase, not less than 70%, in food production and supply will be required to meet the future demand (Braun, 2010). Sustainable development of agricultural production by breeding more productive cultivars using both traditional breeding (Yuan, 2001) and genetic engineering (Qaim, 2010), and by increasing productive potential of existing cultivars through the use of pathogen-free plants (Wintermantel et al., 1997), will hopefully meet this demand.

Yam (*Dioscorea spp.*) is a monocotyledonous climbing crop species that produces edible underground tubers and is propagated vegetatively through the use of tuber setts. It is an economically important food crop in many tropical countries with annual global production put at 73.01 million tones. Nigeria is currently the largest producer with annual production of 47.9 million tones accounting for 67% of global production (FAO, 2017). In some communities in Nigeria, yam is perceived as a religious, social, and cultural crop and is the most favorite food in social functions such as marriage, burials and other traditional ceremonies and rituals. This perception largely explains why ‘New Yam Festivals’ are held annually in these communities (Obidiegwu and Akpabio, 2017). Despite these social and economic importance of yam, its production is severely constrained by the cost and availability of planting material, diseases and pests. Pests and diseases caused by insects, nematodes, fungi, bacteria and viruses, singly or in combination have direct adverse effect on the yield of the crop (Degras, 1993; Hughes et al., 1997; Kenyon et al., 2001). Of these, viruses constitute a major pathological problem in yam production in all growing regions of the world, and are of substantive economic importance not only because of yield losses they cause, but also due to the high cost of preventive measures (Degras, 1993) and restriction of international exchange of germplasms (Hughes et al., 1997). The different groups of viruses infecting yam include *Potyvirus (Yam mosaic virus* (YMV), *Dioscorea alata virus (DAV),* and *Dioscorea dumetorum vi*rus (DdV)); Badnavirus (*Dioscorea alata* bacilliform virus (DaBV)), Cucumovirus (*Cucumber mosaic virus* (CMV)) and Comovirus (*Dioscorea mottle virus* (DMoV)). Apart from DaBV, which is transmitted by mealybugs (*Planococcus citri*), all the other viruses are transmitted by aphids. Studies have shown that YMV, DAV, DaBV, and CMV are mechanically transmissible between yam plants (Kenyon et al., 2001).

Of these viruses, *Yam mosaic virus* (YMV) is the most important virus infecting both cultivated and wild yam species worldwide. The virus is transmitted by aphids in a non-persistent manner, as well as through infected plant material from generation to generation. In nature, YMV has been found in several species of Dioscoreaceae and can be mechanically transmitted to *Nicotiana benthamiana, N. megalosiphon* and *Chenopodium amanticolor* (Odu et al., 2004). Infected plants usually show inter-veinal mosaic, curling, molting and stunted growth, and the disease can result in significant yield losses in yam (Thouvenel and Dumont, 1990; Adeniji et al., 2012). It thus becomes crucial to get resistant or at least virus-free planting materials for farmers. Regrettably, efforts to develop yam seedlings free of yam mosaic virus by conventional breeding methods have been deterred by a number of factors such as long growth cycle, dioecy, poor to no-flowering nature of the plant, polyploidy, vegetative propagation, heterozygous genetic background and poor knowledge of the organization of crop diversity (Mignouna et al., 2008), which prevent the transfer of desirable agronomic traits into the crop. Thus, the use of cryogenic techniques has become a valuable alternative.

Cryotherapy is the treatment of infected plant materials for a short time in liquid nitrogen to rid the plant of the infection based on cryopreservation techniques (Wang and Valkonen, 2009; Wang et al., 2009; Feng et al., 2012). Several plant pathogens including *Cucumber mosaic virus* and *Banana streak virus* (Heliot et al.*,* 2002), *Grape virus A* of grapevine (Wang et al., 2003), *Plum pox potyvirus* in *Prunus spp.* (Brison et al., 1997), *Potato leaf roll virus* in potato (Wang et al., 2006), *Sweet potato feathery mottle virus* and *Sweet potato chlorotic stunt virus* (Wang and Valkonen, 2008), Sweet Potato Little Leaf Phytoplasma in sweet potato (Wang et al., 2008) and Huanglongbing Bacterium in citrus (Ding et al., 2008), have been eradicated using cryotherapy. In yams, cryotherapy of shoot apices and axillary bud, for the purpose of viral eradication, has received wide attention in recent years (Hong et al., 2009; Mandal and Ahuja-Ghosh, 2007; Mandal et al., 2008; Mandal and Dixit-Sharma, 2007; Shin et al., 2013). However, there is no report showing the use of cryotherapy to eradicate YMV in *D. rotundata*. Here, we report for first time the production of YMV-free *Dioscorea rotundata* plants by Cryo-treatment of Axillary buds.

## 2 Materials and methods

### 2.1 Development of *in vitro* plantlets and detection of *Yam mosaic virus* in parent plant

Plantlets of *Dioscorea rotundata* accession TDr 2269 containing YMV were multiplied and maintained *in vitro* as shoot cultures by cultivating nodal segments on yam basic medium (YBM), which is MS basal salts and vitamins (Murashige and Skoog, 1962) containing 0.05 mg/L 6-Benzylaminopurine (BAP), 0.02 mg/L Naphthalene acetic acid (NAA), 25 mg/L Ascorbic acid, 30 g/L sucrose and solidified with 3 g/L gelrite.

Total RNA was extracted from 100 mg leaves of the *in vitro* cultures using RNeasy® Plant Mini Kit by QIAGEN GmbH, Germany. cDNA was synthesized from 1 µg of total RNA using iScript cDNA Synthesis Kit (BIO-RAD Laboratories, Inc.) according to the manufacturer's instructions. One micro liters of the cDNA was then used for PCR to detect 161 bp of YMV-CP gene using the primers: YMV-CP5P: GTGGACAATGATGGACGGTG and YMV-CP3P: CGTATCGGGGCATATACGGT. PCR was performed in a 20 µl reaction volume containing 1 µl genomic DNA (100 ng/µl), 0.1 µl of Ex Taq DNA polymerase, 2 µl of Ex Taq buffer, 1.5 µl of dNTP mix, 1 µl of 10 µM of each primer, and 13.4 µL nuclease-free water. PCR amplification conditions were performed as follows: initial denaturation step at 94 °C for 3 min, followed by 34 cycles of denaturation at 94 °C for 30 s, annealing at 62 °C for 30 s, extension at 72 °C for 1 min, and final extension at 72 °C for 5 min. After amplification, 10 µl of PCR product was resolved on 1% agarose gel stained with gel red.

### 2.2 Axillary bud enlargement, cryo-treatment and regeneration of plantlets

Nodal explants containing axillary buds were excised from *in vitro* cultures of YMV-infected TDr 2269 confirmed by RT-PCR, and cultivated for 6 days on shoot bud induction medium (SBM) consisting of MS salts and vitamins supplemented with 20 g/L sucrose, 10 mg/L BAP, 0.318 mg/L copper sulfate and solidified with 3 g/L gelrite. The pH of the medium was adjusted to 5.8 prior to autoclaving. The enlarged axillary buds were excised and pre-cultured in 10% sucrose of MS liquid medium for 16 h. The buds were then encapsulated in sodium alginate (3% w/v) and hardened in 0.1 M calcium chloride solution to form beads. The beads containing the buds were then washed 3 times in sterile distilled water and placed in liquid MS medium containing 17% sucrose and 2 M glycerol for 1 h. The beads were dried in sterile laminar flow bench for 5 h (dessication). Next, the beads were placed in 2 ml sterile polypropylene cryotubes and incubated in liquid nitrogen for 1 h. The control group was plated directly in recovery medium. Thereafter, the cryotubes were retrieved from liquid nitrogen and immersed in a water-bath at 40 °C for 5 min. The beads were then transferred to a regeneration medium (MS salts and vitamins supplemented with 20 g/L sucrose, 1 mg/L BAP, 0.318 mg/L copper sulfate, 3 g/L gelrite, pH 5.8) and sub-cultured every 2 weeks until shoots developed (Manoharan et al. 2016). Regenerated shoots were excised and transferred to MS medium (salt and vitamins) supplemented with 30 g/L sucrose, 0.02 mg/L NAA, 0.05 mg/L BAP, 25 mg/L Ascorbic acid, pH5.8 and 3 g/L gelrite as gelling agent.

### 2.3 Screening of regenerated plantlets for the presence of YMV by RT-PCR

RNA was extracted from 100 mg leaves of regenerated shoots using RNeasy® Plant Mini Kit and cDNA synthesized using PrimScript™ RT reagent Kit with gDNA Eraser (Perfect Real Time). Two micro liters of the cDNA synthesized was used for PCR to detect 161 bp of YMV-CP gene using the primers: YMV-CP5P and YMV-CP3P. Yam ubiquitin gene was used as internal control to check the quality of the cDNA using the following primers, Yam_Ubq: ATGAGGAACCAATGGCTGAG (forward) and TTGCCAGCATGTCTATCTGC (reverse). The reaction was constituted in a total of 50 µL volume containing 5 µL of 10X Ex Taq buffer (1X), 5 µL dNTPs (200 µM), 1 µL of 10 µM YMV-CP-5P (0.2 µM), 1 µL of 10 µM YMV-CP-3P (0.2 µM), 0.40 µL of Ex Taq polymerase (1.25 units), 2 µL of cDNA and 35.60 µL of nuclease-free water. PCR reaction and condition were same as described earlier in Section 2.1. After amplification, 10 µL of PCR product was resolved on 1% agarose gel stained with gel red.

### 2.4 *In vitro* multiplication of YMV-free plants and establishment in greenhouse

Ten lines of YMV-free plants confirmed by RT-PCR and YMV-infected control plants were multiplied and six plants per line were subsequently transferred to the greenhouse for testing. For shoot induction, nodal explants containing axillary buds were excised and cultured on yam basic medium (YBM). The pH of the medium was adjusted to 5.8 before autoclaving. Rooted plantlets were transferred to the greenhouse in pots containing garden soil and covered with transparent polybags for 14 days for acclimatization. The temperature was maintained at 25 °C ± 2 °C, the relative humidity was 70%.

### 2.5 Evaluation of regenerated plants in greenhouse

The established YMV-free plants (10 lines, 6 plants per line) and the control (6 plants) in the greenhouse were assessed every day for mosaic symptoms development but the final classification was recorded 17 weeks after transfer to the soil. The number of plants and number of leaves per plant showing mosaic symptoms were recorded. Also, plant height, number of leaves, number of mini tubers, and weight of mini tubers were recorded 17 weeks after transfer to the soil. Data were collected from all six plants per line and subjected to analysis of variance (ANOVA). Means were separated using Least Significant Difference (LSD) test.

### 2.6 Evaluation of established plants for virus accumulation by real time RT-PCR

RNA was extracted from leaves of twelve weeks old plants and cDNA synthesized as described in Section 2.3. The cDNA synthesized were subjected to Real Time RT-PCR using the primers: YMV-CP_qPCR: CAGATATGCGTTCGACTTCTTA (forward) and AGGCTGTGCATGTCTTTC (reverse); Ubq_qPCR: CAGTCATGGTGCGATGTT (forward) and CCTCACAACTTCCAAGAGTTC (reverse) as internal control. Real Time PCR was performed in Applied Biosystems StepOne™ and StepOnePlus™ Real Time PCR system (Applied Biosystems, Japan Ltd.) using KOD SYBR® qPCR Mix (TOYOBO Company Limited, Japan). The Real Time PCR reaction was constituted in a total of 20 µL: 7.52 µL distilled water, 10 µL KOD SYBR qPCR mix (1 X), 0.04 µL forward primer (0.2 µM), 0.04 µL reverse primer(0.2 µM), 0.4 µL 50X ROX reference dye (0.1 X). The PCR cycling conditions were as follows: pre-heating at 98 °C (2 min), followed by 40 cycles of 98 °C (10 s .), 55 °C (10 s .), 68 °C (30 s .), with melting curve analysis (60–99 °C), for both YMV-CP and Ubq. Ct values of the standard were used to generate calibration curves for both YMV-CP and Ubq. Sample Ct values were then interpolated in the calibration curve to determine the expression level of both YMV-CP and Ubq. The relative expression of YMV-CP was calculated after normalization of YMV-CP expression using ubiquitin as reference gene (Livak and Schmittgen, 2001).

## 3 Results

### 3.1 Detection of *Yam mosaic virus* in parent plant

**Fig. 1:**
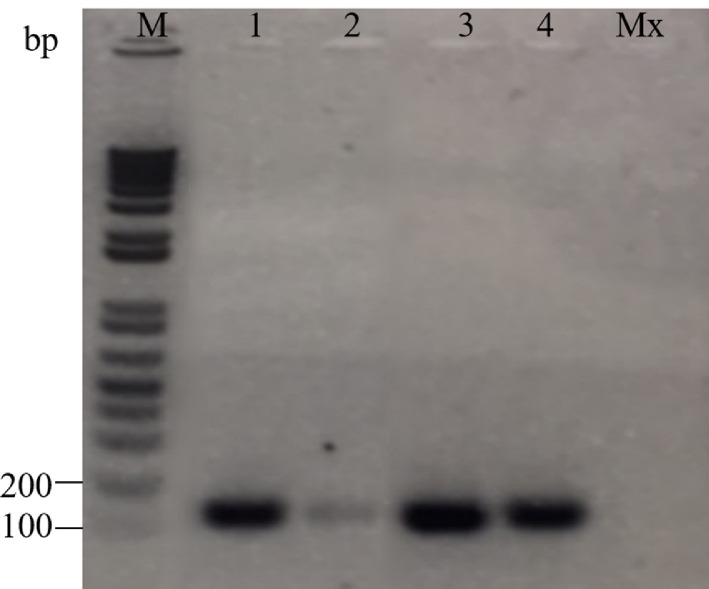
ART-PCR analysis of YMV-CP transcript from YMV-infected plants by RT-PCR. M, DNA marker; 1–4, leaf samples from four different plants; Mx, PCR master mix.

RT-PCR analysis using *Yam mosaic virus* coat protein (YMV-CP) gene was carried out to confirm the presence of the virus in the parent plant before cryo-treatment. The result is presented in Fig. 1. From the analysis, 161 bp of YMV-CP was amplified in all the samples tested, indicting the presence of YMV in the parent plant.

### 3.2 Plantlet regeneration after cryo-treatment

**Fig. 2:**
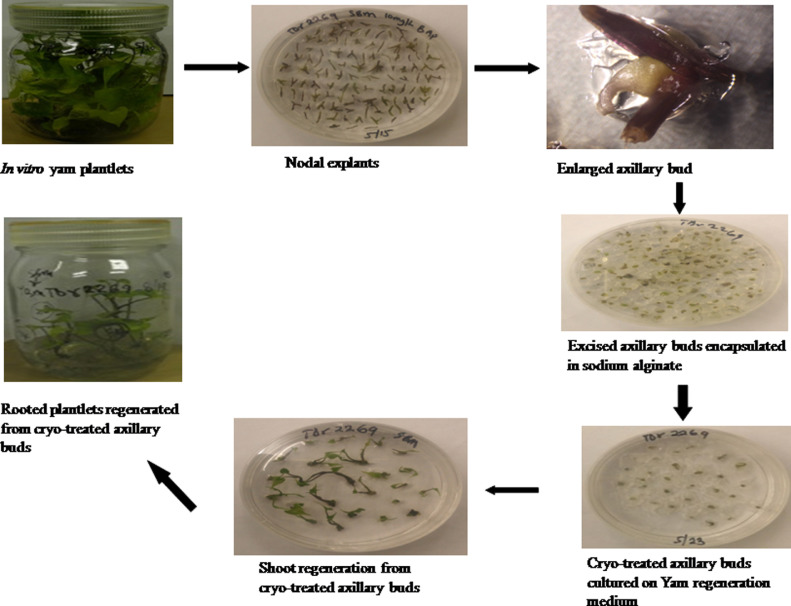
Process of cryo-treatment and plant regeneration.

Axillary buds excised from YMV-infected plants confirmed by RT-PCR were cryo-treated and plantlets were regenerated accordingly (Fig. 2). A regeneration frequency of 76.33% was obtained from cryo-treated buds as against 95% recorded from the untreated buds (Table 1).

**Table 1:** Regeneration (%) and YMV eradication (%) of control and cryo-treated TDr 2269 axillary buds.

Treatment group	Regeneration (%)^a^	YMV eradication (%)^b^
Non-cryotreated (LN−)	19/20 (95)	0/10 (0)
Cryo-treated (LN+)	42/55 (76.33)	20/20 (100)

a Explants developing leafy shoots and roots.

b YMV-free *in vitro* plants after RT-PCR analysis.

### 3.3 Yam mosaic virus detection by RT-PCR

**Fig. 3:**
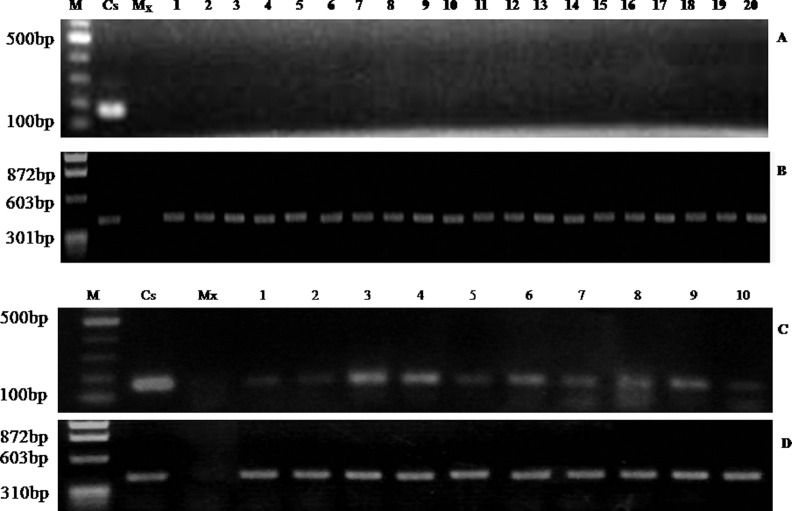
Detection of YMV-CP in regenerated plants. (A) RT-PCR of plants regenerated from cryo-treated buds using YMV-CP specific primers; (B) Yam ubiquitin; (C) RT-PCR of plants regenerated from non-cryotreated buds using YMV-CP specific primers; (D) Yam ubiquitin; M – DNA marker; C_s_ – YMV-infected stock plant; Mx – PCR master mix; 1–20 – plants regenerated from cryo-treated axillary buds; 1–10 – YMV-infected plants regenerated from non-cryotreated axillary buds.

To determine whether cryo-treated regenerated plants were YMV-free, RT-PCR was carried out. The result showed no amplification of the 161 bp fragment of YMV-CP in all the 20 plants sampled from plants regenerated from cryo-treated buds (Fig. 3, Table 1). This indicates that they were YMV-free. In contrast, YMV-CP gene was amplified in all the ten sampled plants regenerated from the non-treated buds, indicating that none was free of YMV (Fig. 3, Table 1).

### 3.4 Evaluation of regenerated plants in the greenhouse

**Fig. 4:**
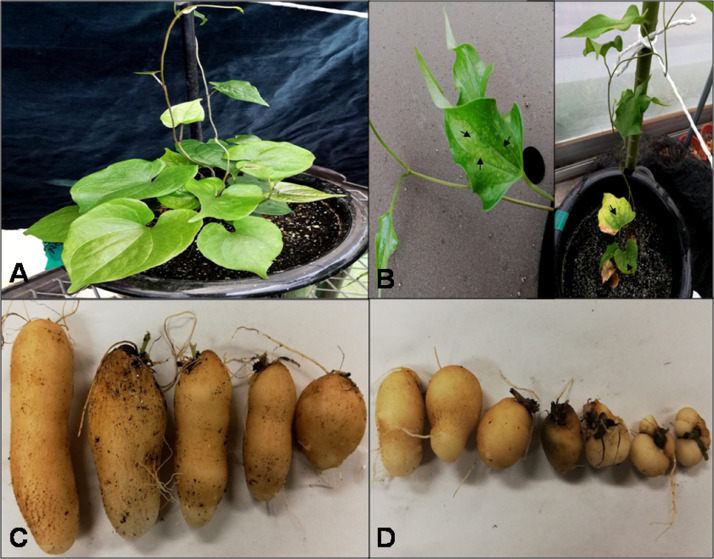
Performance of Cryo-treated plants in the greenhouse. (A) Yam plants from cryo-treated bud showing no mosaic symptoms under greenhouse conditions; (B) Yam plants from non-cryo-treated bud showing mosaic symptoms under greenhouse conditions; (C) Mini-tubers produced by plants regenerated from cryo-treated buds under greenhouse conditions; (D) Mini-tubers produced by plants regenerated from non-cryo-treated buds under greenhouse conditions.

To further screen cryo-treated plants, YMV-free yam plantlets confirmed by RT-PCR were propagated in the greenhouse until minitubers were produced. None of the plants regenerated from cryo-treated buds showed mosaic symptom (Fig. 4(A)) 17 weeks after culture. In contrast, control plants started developing mosaic symptoms from the 8th day after transfer to the greenhouse. At the end of the evaluation period (17 weeks), all the control plants (100%) had developed mosaic symptoms (Fig. 4(B)). Vine length was significantly reduced (*p* < 0.05) in non-treated control plants (8.95±0.46 cm), whereas plants in line C20 (cryo-treated) had the longest vine length (26.56±7.04 cm) Table 2). Plants in line C5 (cryo-treated) produced more mini-tubers (1.50±0.23) as against 1.00±0.00 recorded for the control, C1, C2, C8, C10, C13 and C20 (Table 2). The weight of mini-tubers in the cryo-treated plants were significantly (*p* < 0.05) higher, ranging from 6.31±1.33 g in C1 to 20.48±3.11 g in C20 (Fig. 4(C), Table 2), compared to those in the control which were only 1.91±0.39 g (Fig. 4(D), Table 2). Altogether, Cryo-treated plants produced more and larger tubers (Fig. 4(C)) than the control plants (Fig. 4(D)).

**Table 2:** Performance of control and cryo-treated TDr 2269 plants under greenhouse conditions.

Trmt group	Number of plants with mosaic symptoms	Vine length (cm) X ± S.E	Number of mini tubers produced X ± S.E	Weight of mini tubers (g) X ± S.E	Number of leaves X ± S.E
CTRL	6	8.95^cde^±0.46	1.00^c^±0.00	1.91^fg^±0.39	8.16^NS^±0.83
C1	0	14.93^bcde^ ±3.47	1.00^c^ ±0.00	6.31^de^±1.33	11.33 ^NS^ ±2.34
C2	0	14.08^bcde^ ±2.77	1.00^c^ ±0.00	12.38^bcdef^±2.25	11.00 ^NS^ ±1.21
C5	0	19.35^bcde^ ±3.84	1.50^a^±0.23	13.10^bcde^±2.27	8.33 ^NS^ ±1.14
C8	0	14.26^bcde^ ±2.06	1.00^c^±0.00	12.53^bcdef^±1.19	10.50 ^NS^ ±1.47
C9	0	13.55^bcde^ ±1.98	1.16^bc^±0.16	13.66^bcde^±2.38	8.66 ^NS^ ±1.14
C10	0	14.08^bcde^ ±3.30	1.00^c^ ±0.00	8.78^cdef^±1.38	8.16 ^NS^ ±1.74
C11	0	20.68^abcd^ ±3.83	1.16^bc^±0.16	15.38^bcd^±1.976	9.50 ^NS^ ±1.50
C13	0	9.01^cde^ ±1.18	1.00^c^±0.00	9.56^bcde^±1.55	5.33 ^NS^ ±0.42
C18	0	14.28^bcde^ ±3.47	1.33^ab^±0.21	11.21^bcde^±4.26	9.16 ^NS^ ±2.88
C20	0	26.56^abc^ ±7.04	1.00^c^±0.00	20.48^ab^±3.11	9.66 ^NS^ ±1.74

Lower case letters denote differences among the means on the basis of LSD test. NS indicates means with no significant differences (*p* > 0.05).

### 3.5 Determination of virus accumulation by real time RT-PCR

**Fig. 5:**
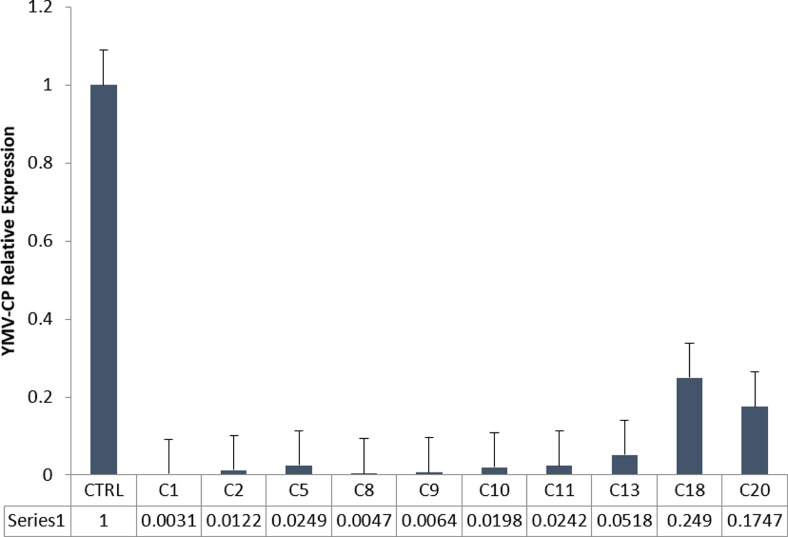
YMV-CP relative expression in cryo-treated and control plants. Plants regenerated from YMV-infected non-cryotreated buds (CTRL); Plants regenerated from cryo-treated buds (C1–C20).

Cryo-treated and non-treated yam plants established in the greenhouse were evaluated for virus accumulation in the leaves by real time RT-PCR. The result indicated significantly higher accumulation of YMV which is correlated with high relative expression of YMV-CP in the leaves of non-treated yam plants compared to cryo-treated plants (Fig. 5). There was no expression in lines C1–C13 which had expression levels ranging from 0.0031 in C1 to 0.05 in C13. Lines C18 and C20 had very little expression (Fig. 5), indicating the effectiveness of the cryo-technique used in this study.

## 4 Discussion

Pathogen-induced diseases have for a long time threatened the sustainable production of agricultural and horticultural crops (Wintermantel et al., 1997; Wang et al., 2011). Several crop species that ensure food security in many different parts of the world are vegetatively propagated and therefore particularly prone to losses caused by viruses that are transmitted from generation to generation in the planting materials (Hadidi et al., 1998; Loebenstein et al., 2001; Loebenstein and Thottapilly, 2003). The availability of pathogen-free planting materials is crucial for high yields and quality of these crops. Virus eradication methods such as meristemming, viral drug therapy, thermotherapy, electrotherapy, and cryotherapy, singly or in combination have been employed over the years with varying levels of success. In cryotherapy, virus-infected cells are eliminated by the lethal effects of ultra-low temperature; no mechanical removal of tissues is necessary. Cryotherapy takes advantage of physio-anatomical differences between meristematic and other tissues, and is carried out to destroy the differentiated, infected cells by a brief cryo-treatment in liquid nitrogen (Wang and Valkonen, 2009).

It has been shown by light-microscopy observation that freezing in liquid nitrogen kills the cells with large vacuoles in the lower layers of the meristem, while leaving a few top layers of dense cells alive (Brison et al., 1997; Wang et al., 2008; Wang and Valkonen, 2008; Wang et al., 2009; Wang and Valkonen, 2009). When shoot tips are subjected to freezing in liquid nitrogen, large cells with bigger vacuoles which contain more water and are more likely to be infected by the virus, are killed. Only small cells with dense cytoplasm, which are located in the top layers of the meristem and are likely to be virus-free, survive freezing in liquid nitrogen (Helliot et al., 2002; Wang et al., 2003; Wang and Valkonen, 2008). This is thought to provide an explanation for why plants regenerated from cryotherapy can be freed from virus.

In this study, eradication of YMV from Tropical *Dioscorea rotundata* by cryotherapy was effective. It was however observed that the regeneration level after cryotherapy was slightly lower compared to the non-cryotreated plants. This difference was compensated by the higher proportion of virus-free regenerants obtained following cryotherapy, this is similar to reports by Helliot et al. (2002), Wang et al. (2003, 2006) and Ding et al. (2008). In contrast, Shin et al. (2013) reported different regeneration percentage and YMV eradication percentage in *D. opposite* following cryo-treatment of the shoot apices. The reasons for this difference in virus elimination efficiency are not known, although factors such as difference in cryo-treated plant parts as well as the different dehydration protocol, plant cultivar, and, perhaps, virus strain may have had a bearing in determining it.

The cryo-treated buds produced healthy plantlets following *in vitro* propagation and showed high level of acclimatization on transfer to the greenhouse. This indicates that the cryo-treatment did not interfere with the growth and general performance of the plants *in vitro* and under greenhouse conditions. Although, evaluation of regenerated plantlets with RT-PCR revealed complete elimination of YMV from yam plantlets from cryo-treated buds, re-evaluation of the yam plants after greenhouse acclimatization with qPCR showed the accumulation of YMV in lines C18 and C20 at a reduced concentration. This indicates that YMV was not completely eliminated in these lines although leaf mosaic symptoms were not present, probably due to the fact that the viral titer was greatly reduced to a negligible concentration. Furthermore, qPCR is a more sensitive technique than RT-PCR and so it was able to detect very little accumulation of the virus. This is similar to reports by Ding et al. (2008). The implication of the result obtained in this study is that the limitations faced with international germplasm exchange of *Dioscorea spp.* due to viral contamination will be eliminated as virus-free germplasms can be produced.

Several viruses have been reported to infect yams including members of the family Potexviridae; Potyviridae; Bromoviridae; Caulimoviridae (Hughes et al., 1997; Odu et al., 1999). The effectiveness in the elimination of YMV by cryo-treatment of the buds can be adopted for other yam viruses. This may be achievable due to the fact that most viruses exhibit similar mechanism of infestation and replication in host tissues; and the fact that crytotherapy has been reported to be effective in eliminating most viruses, except for a group of viruses that can infect meristematic cells which can be eliminated using combined thermotherapy and cryotherapy (Gallard et al., 2011).

It has been reported that yam tuber yield depends on the ability of healthy yam leaves to efficiently trap, convert and sink the sun's light energy into chemical energy in tubers during photosynthesis (Nweke et al., 1991). In the present study, reduction in tuber size and quality in YMV-infected control plants could be said to be due to the varying shades of mosaic and chlorotic leaf discoloration and malformation symptoms. This invariably resulted in the reduction of photosynthetic efficiency of the infected plants and consequently the reduction in tuber yield and quality (Amusa et al., 2003; Eni et al., 2013).

## 5 Conclusion

Our results demonstrate that YMV can be effectively eliminated from Tropical *Dioscorea rotundata* by cryotherapy of the axillary buds of infected stock plants. The cryo-treatment did not interfere with regeneration, growth and general performance of the plants *in vitro* and under greenhouse conditions. To establish the economic viability of this approach, extensive study of field performance of the regenerated plants will be necessary.

## CRediT authorship contribution statement

**Effiom Eyo Ita:** Writing - original draft. **Edak Aniedi Uyoh:** Writing - original draft, Supervision. **Ikuo Nakamura:** . **Valentine Otang Ntui:** Writing - original draft, Conceptualization, Supervision.

## Declaration of Competing Interest

The author declare that no conflict of interest exist.
